# Getting health information to internally displaced youth in Afghanistan: can mobile phone technology bridge the gap?

**DOI:** 10.12688/gatesopenres.13008.2

**Published:** 2019-10-23

**Authors:** Sayed Omar Alami, Lisa Dulli, Leila Dal Santo, Sayed Haroon Rastagar, Sediq Seddiqi, Shafiqullah Hemat, Jane Machlin Burke, Catherine S. Todd

**Affiliations:** 1 Afghanistan Country Office/HEMAYAT Project, FHI 360, Kabul, Afghanistan; 2 Global Health and Population Research, FHI 360, Durham, NC, 27701, USA; 3 Reproductive, Maternal, Newborn and Child Health Division, FHI 360, Durham, NC, 27701, USA; 4 Assess, Transform, Reach Consulting, Kabul, Afghanistan; 5 Health Promotions Department, Ministry of Public Health Islamic Republic of Afghanistan, Kabul, Afghanistan

**Keywords:** Afghanistan, adolescents, mobile phone, information and communications technology for health, family planning, maternal health, child health, social and behavior change communication

## Abstract

**Background:** Afghanistan ranks among the most disadvantaged globally for many key reproductive, maternal, newborn and child health (RMNCH) indicators, despite important gains in the past decade. Youth (15 to 24 years) are a key audience for RMNCH information as they enter adulthood, marry and begin families; however, reaching Afghan youth with health information is challenging. Internally displaced persons (IDPs), including youth, experience additional challenges to obtaining health-related information and services. This study measured current and preferred RMNCH information channels to explore the feasibility of using mobile phone technology to provide RMNCH information to IDP youth in Afghanistan.

**Methods:** We conducted a sub-group analysis of survey data from a mixed-methods, cross-sectional, formative assessment to understand current access to RMNCH information. The target population for this analysis includes 15-25-year-old male and female IDP youth from three Afghan Provinces. Survey data were collected using a structured questionnaire administered through face-to-face interviews. Data were analyzed descriptively.

**Results:** A total of 450 IDP youth were surveyed in the three provinces (225 male and 225 female). Access to RMNCH information outside of health facilities was limited. Mobile phone ownership was nearly universal among male participants, yet considerably lower among females; nearly all participants without personal phones reported access to phones when needed. Although few participants spontaneously mentioned mobile phones as a preferred source of RMNCH information, most male and female respondents reported they would be very or somewhat likely to use a free, mobile-phone-based system to access such information if offered.

**Conclusions:** Given widespread access and considerable interest voiced by participants, mobile phones may be a viable way to reach IDP youth with important RMNCH health information in this fragile setting. Interventions should be designed and pilot-tested to identify the most appropriate platforms and information content and to further document feasibility and acceptability.

## Introduction

Despite improvements over the past two decades in reproductive, maternal, newborn and child health (RMNCH), Afghanistan continues to rank among the most disadvantaged countries globally for many health indicators ^1, 2^. Child and maternal mortality remain critical public health issues, with higher rates in rural settings compared to urban ^3^. Though some newborn and child health statistics have improved, immunization and nutrition indicators lag behind other countries regionally and vary markedly ^3– 5^ between provinces and urban and rural settings. Total fertility rate (TFR) among Afghan women, which contributes to maternal mortality, is also high at 5.3 children per woman, and age at first birth is low (median 20.1 years) with 12% of girls ages 15–19 years initiating childbearing ^3^. Modern contraceptive method use to space or limit pregnancies has stagnated over the last decade, with only 20% of married women reporting modern method use ^3^.

Afghanistan has a “youth bulge” with more than half of its population younger than 19 years; youth aged 15 to 24 years comprise 22.2% of the population ^6^. As these youth enter adulthood, marry, and begin their families, they constitute a key target for RMNCH interventions aimed to improve health and reduce maternal and child mortality. They are also an important audience for health-related information, particularly around RMNCH. However, reaching Afghan youth with needed RMNCH information is challenging, as multiple barriers limit young peoples’ access to formal health services ^7, 8^.

Internally displaced persons (IDPs), including youth, experience additional challenges to obtaining essential health-related information and services. Years of armed conflict, natural disasters like drought and flooding, and widespread poverty have led to large-scale migration of intact family units both within and from Afghanistan ^9– 11^. As of 2018, nearly 2.6 million of the estimated 35.5 million people in Afghanistan were internally displaced ^9, 10^. The factors that contribute to internal displacement also reduce health service access. A 2018 United Nations Refugee Agency (UNHCR) report documented that up to 42% of Afghan IDPs are unable to access health care, with those in urban areas having slightly better access than those in rural settings ^12^. Key reasons for limited health care access were high cost and perceived low quality of available healthcare ^12^.

More information is needed on alternative ways to reach displaced youth with health information, particularly in fragile settings. Several programs have succeeded in reaching various audiences with health information through platforms that use mobile phone technology across multiple global contexts ^13– 17^. The exponential growth of mobile phone technology has created opportunities to connect people with information at a scale previously unfathomable and Afghanistan is no exception. Mobile phone coverage is increasing within Afghanistan, in terms of number of users and use among target populations, including women and youth. In 2014, Afghanistan had an estimated 12 million mobile phone subscribers, and a 2018 estimate reported subscriptions at 22.0 million ^18, 19^. A 2012 survey of Afghan women revealed that 80% had mobile phone access: 48% of women owned a mobile device and 32% could borrow one when needed ^20^.

To optimize exposure to RMNCH-related information and programming to create demand for health services, a greater understanding is needed of current exposure to and preferences for information channels and content among critical target audiences, such as IDP youth. The objective of this study was to measure current and preferred RMNCH information channels, content and media preferences, and to explore the feasibility of using mobile phone technology to reach IDP youth in Afghanistan with RMNCH information.

## Methods

### Study background

We conducted a sub-group analysis of survey data collected in a mixed-methods, cross-sectional, formative assessment to inform programming for the United States Agency for International Development-funded Helping Mothers and Children Thrive in Afghanistan (HEMAYAT) project ^21^. The assessment was designed to gather information from youth 15–25 years and adult men >25 years of age to identify health information and service gaps related to RMNCH outcomes and inform content and channel selection to segments of these populations, including IDPs, for targeted programming. For these analyses, we include data from a convenience sample of female and male IDP youth, ages 15 to 25 years. Youth who self-identified as IDP were recruited from households located in areas with large IDP populations in Kandahar, Nangarhar, and Takhar Provinces, selected based on their geographic and ethnic diversity. Survey data were collected March–July 2017 using a structured questionnaire administered through face-to-face interviews conducted by trained research assistants. Data used for these analyses include participant background information, household possession of media devices (i.e. television, radio and mobile phones), exposure to health information from various channels, desires for various types of health information and preferred channels of communication, as well as reported likelihood to use a free mobile phone-based system to receive health information.

Geographic areas were selected based on guidance from UNHCR, the Ministry of Refugees and Repatriation, and the Danish and the Norwegian Refugee Committees, who have pre-existing relationships with IDP communities and their leaders. Households in these communities were sampled using a random walk technique ^22^. The approach used for this random-walk sample involved selecting a central community landmark, such as a mosque, then selecting one house at random to be the starting point. From there households were approached at regular intervals (e.g. every 3rd). Household selection continued in each area until reaching the requisite sample size. Verbal informed consent was obtained from eligible youth interested in participating then a structured questionnaire was administered by sex-matched data collectors in a private setting within the house.

In each province, 150 IDP youth (75 male and 75 female) were recruited, resulting in 450 IDP youth across the three study provinces. Equal samples of male and female youth were selected to examine differences by sex. Samples sizes were calculated based on the ability to detect a minimum of a 20 percentage-point difference from a baseline of 40% in various indicators for each sub-population, assuming 90% power and an alpha of 0.05; further details on sample size calculations and sampling are available in the project report ^21^. Data were analyzed descriptively and stratified by province and sex. Complete case analysis was conducted with numbers of missing data for each variable reported in tables.

### Ethical approval

The study was approved by the Protection of Human Subjects Committee of FHI 360 (# 844213) and the Afghanistan Ministry of Public Health Institutional Review Board (#355310) prior to implementation.

## Results

A total of 462 IDP youth were recruited to participate in the survey. Ten of the 462 declined participation (2.2%); one person consented but refused to answer the remaining survey questions and data from one further participant was dropped because of incomplete interviews, leaving 450 total study participants from Kandahar, Nangarhar, and Takhar Provinces, including 225 male and 225 female youth. Characteristics of non-responders are not available as once a person declined to participate, no further information was recorded.

Just over half of female IDP youth were married, compared to 39% of their male counterparts ( Table 1). Nearly all participants were born in Afghanistan. One-third of male and nearly half of female IDP youth reported currently living in their birth province. About two-thirds of male IDP youth had any formal education, compared to just under one-fifth of female youth; education levels varied markedly by province ^22^.

**Table 1.** Background characteristics of study participants, by sex and province, among IDP youth in Afghanistan, 2017.

Media	Kandahar		Nangarhar		Takhar	
	Men (n=75) %	Women (n=74) %	Men (n=75) %	Women (n=76) %	Men (n=75) %	Women (n=75) %
Age in years (mean)	20.3	21.6	20.1 ^1^	22.1	22.7 ^1^	21.6
Married	24.0	21.6	26.7	64.5	66.7	70.7
Highest level of education						
None	28.0	90.5	28.0	97.4	52.0	57.3
Primary	10.7	2.7	16.0	1.3	25.3	18.7
Secondary	14.7	1.4	14.7	1.3	13.3	8.0
Higher	45.3	5.4	40.0	0.0	6.7	16.0
Vocational	1.3	0.0	1.3	0.0	2.7	0.0
Able to read a full sentence	60.0	5.4	50.7	0.0	17.3	9.3
Born in Afghanistan	94.7	96.0	85.3	89.5	93.3	92.0
Living in province of birth	62.7	56.8	16.0	51.3	21.3	37.3
Received health care from medical provider in prior 6 months	46.7	50.0	36.0	77.6	28.0	46.7

^1^1 missing

### Media access and exposure

Reported household radio and television ownership differed by province, but were similar by sex, with about half reporting a radio and one-fourth reporting a television in their households Table 2; reported television ownership was largely concentrated in Kandahar ( Table 2). Mobile phone ownership and access differed considerably by sex and province. Nearly all male IDP youth (94.0%-98.7%) reported mobile phone ownership but was considerably lower among female respondents overall and lowest in Nangarhar. Having a smartphone in the household and prior internet use were generally low, but lower for females than males.

**Table 2.** Access to media channels in household, by sex and province, among IDP youth in Afghanistan, 2017.

Media	Kandahar		Nangarhar		Takhar	
	Men (n=75) %	Women (n=74) %	Men (n=75) %	Women (n=76) %	Men (n=75) %	Women (n=75) %
Radio in household	68.0	60.8	68.0	59.2	21.3	18.7
Television in household	49.3	50.0	18.7	14.5	14.7	12.0
Basic mobile phone in household	80.0	35.1	70.7	31.6	89.3	94.7
Smartphone in household	49.3	8.1	16.0	10.5	9.3	17.3
Personally owns mobile phone	98.7	55.4	94.7	38.2	98.7	56.0
Able to use a mobile phone (own phone or one that belongs to another)	100.0	100.0	98.7	97.4	98.7	81.3
Ever accessed the internet	18.7	5.4	6.7	0.0	1.3	2.7

Few IDP youth reported any exposure to print media or television, except for television in Kandahar ( Table 3). Just over half of male IDP youth reported at least weekly radio exposure, compared to approximately one-quarter of female IDP youth, varying by province.

**Table 3.** Reported exposure to different media, by sex and province, among IDP youth in Afghanistan, 2017.

Media channel	Kandahar		Nangarhar		Takhar	
	Men (n=75) %	Women (n=74) %	Men (n=75) %	Women (n=76) %	Men (n=75) %	Women (n=75) %
Print (newspaper or magazine)						
None	69.3	94.6	81.3	97.4	92.0	100.0
Daily	1.3	0.0	2.7	0.0	1.3	0.0
Weekly	16.0	1.4	5.3	4.3	1.3	0.0
Less than weekly	13.3	4.1	10.7	1.3	5.3	0.0
Radio						
None	41.3	43.2	12.0	65.8	77.3	85.3
Daily	28.0	51.4	64.0	6.6	12.0	6.7
Weekly	28.0	2.7	22.7	14.5	9.3	5.3
Less than weekly	2.7	2.7	1.3	13.2	1.3	2.7
Television						
None	46.7	48.7	78.7	79.0	85.3	85.3
Daily	41.3	47.3	16.0	10.5	8.0	13.3
Weekly	12.0	4.1	4.0	7.9	6.7	1.3
Less than weekly	0.0	0.0	1.3	2.6	0.0	0.0

Few study participants recalled exposure to health-related information communicated through any channel in the 30 days prior to the survey ( Table 4). Among those who recalled such information, immunization, family planning, hygiene and nutrition were the most frequently reported topics, though responses differed considerably by sex and channel. Few male and no female youth reported receiving health-related information by internet.

**Table 4.** Types of health information received in prior 30 days, by medium and topic, among those with reported receiving any health information by each channel and by sex, IDP youth in Afghanistan, 2017.

	Radio		Television		Internet	
Health Topic ^1^	Male (n=59) %	Female (n=29) %	Male (n=46) %	Female (n=26) %	Male (n=27) %	Female (n=0) %
Immunization	72.9	69.0	67.4	53.9	44.4	--
Influenza	30.5	41.4	30.4	38.5	29.6	--
Family planning	22.0	6.9	50.0	26.9	22.2	--
Hygiene	37.3	37.9	65.2	19.2	44.4	--
Nutrition	25.4	20.7	52.2	15.4	44.4	--
High blood pressure	11.9	20.7	13.0	11.5	33.3	--
Smoking prevention	10.2	13.8	15.2	23.1	25.9	--

^1^More than one response possible, percentages do not add to 100.

### Health information and preferred information sources

Nearly all participants indicated a desire for more health information; health maintenance (e.g., exercise, nutrition) ranked highest among desired topics ( Table 5). Other areas prioritized by male youth included smoking cessation (32.0%), accident prevention, and newborn care (18.2% each). Female youth prioritized depression and mental health issues (29.3%), stress reduction (24.9%), newborn care (22.2%) and family nutrition (22.7%); these topics were mentioned most frequently in Kandahar. Important RMNCH topics including antenatal care, breastfeeding and birth spacing/family planning were lower priorities for both sexes, varying across provinces.

**Table 5.** Type of health information participants would like, by sex and province, among IDP youth in Afghanistan, 2017.

Message content ^1^	Kandahar		Nangarhar		Takhar	
	Men (n=75) %	Women (n=74) %	Men (n=75) %	Women (n=76) %	Men (n=75) %	Women (n=75) %
Health maintenance	49.3	52.7	53.3	39.5	58.7	73.3
Smoking cessation	42.7	18.9	22.7	11.8	30.7	1.3
Accident prevention	18.7	21.6	20.0	34.2	16.0	1.3
Stress reduction	28.0	46.0	5.3	19.7	17.3	9.3
Depression or other mental health issues	25.3	70.3	10.7	9.2	13.3	9.3
Antenatal care	9.3	10.8	4.0	2.6	30.7	10.7
Care during labor and delivery	10.7	12.2	9.3	14.5	21.3	14.7
Newborn care	20.0	24.3	14.7	10.5	20.0	32.0
Breastfeeding	13.3	89.5	8.0	10.5	6.7	0.0
Family nutrition	10.7	44.6	32.0	4.0	6.7	20.0
Birth spacing/FP methods	1.3	13.5	4.0	7.9	6.7	25.3

^1^More than one response possible, percentages do not add to 100.

Youth across provinces strongly preferred health care providers as sources of health information, with home visits by community health workers (CHWs) a distant second ( Table 6). Although few male IDP youth spontaneously mentioned mobile phones as a way to access RMNCH information, 16.4% of female IDPs youth were interested in having such information provided though a telephone hotline number, most markedly in Kandahar.

**Table 6.** Preferred sources of RMNCH information, by sex and province among IDP youth in Afghanistan, 2017.

Channel ^1^	Kandahar		Nangarhar		Takhar	
	Men (n=75) %	Women (n=74) %	Men (n=75) %	Women (n=76) %	Men (n=75) %	Women (n=75) %
Health care provider	81.3	50.0	76.0	59.5	68.0	81.3
Home visit by CHW	29.3	21.5	26.7	38.2	40.0	28.0
Printed information	0.0	1.4	1.3	1.3	5.3	0.0
Television	12.0	6.8	13.3	4.0	6.7	6.7
Radio	12.0	17.6	14.7	5.3	5.3	1.3
Mobile phone voice call	4.0	9.5	1.3	2.6	5.3	6.7
Telephone hotline to call	0.0	24.3	0.0	14.5	0.0	10.7

^1^More than one response possible, percentages do not add to 100.

### Mobile phones as a source of health information

**Figure 1.**
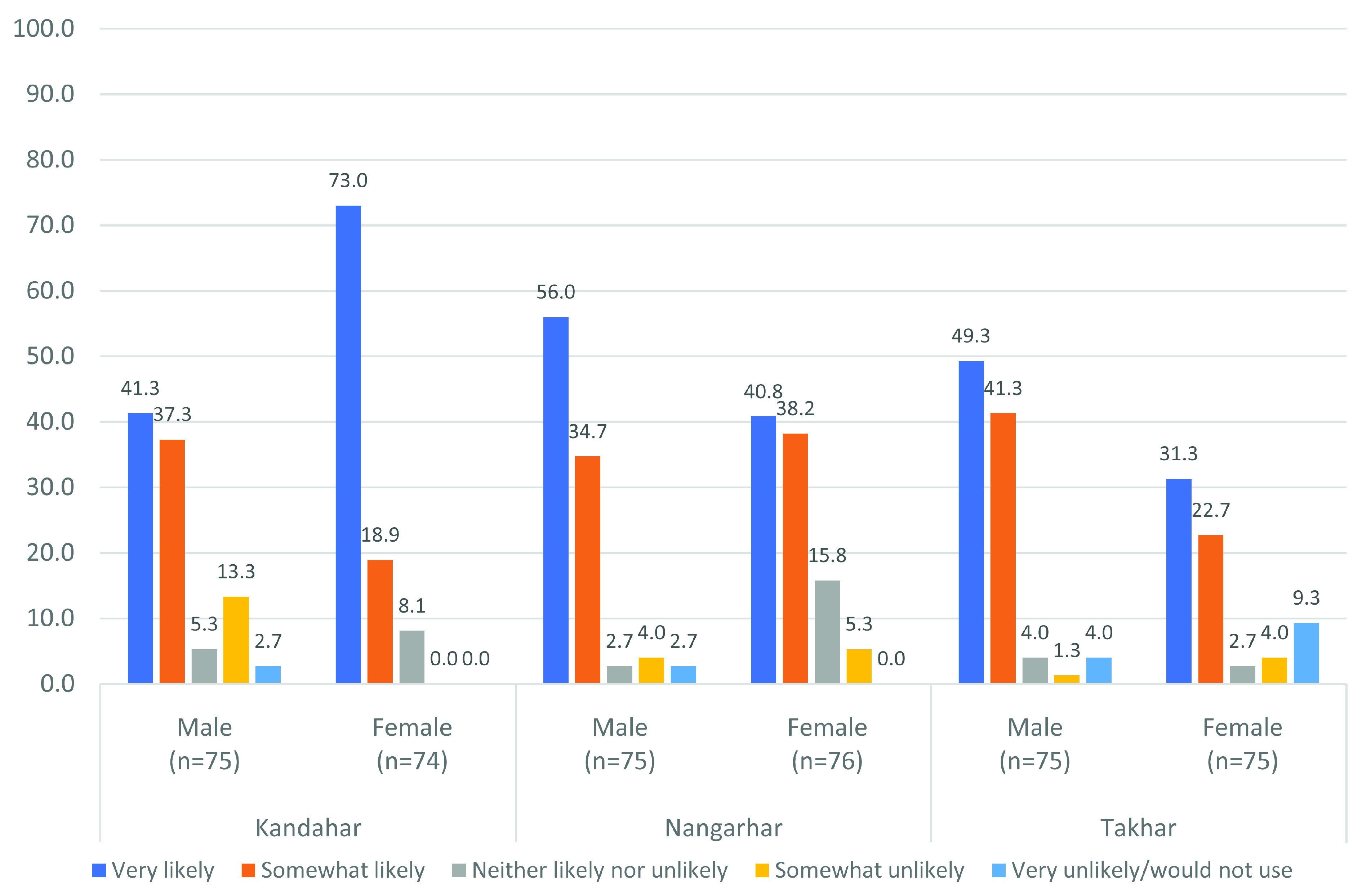
Likelihood of use of free mobile-phone based information on reproductive health among IDP youth, by sex and province, Afghanistan, 2017 (n=450).

IDP youth were asked how likely they would be to use a free mobile phone-based system to receive RMNCH information. Nearly all respondents, regardless of sex or province, said they would be somewhat or very interested in such a system ( Figure 1).

Participants were further asked if the phone-based system should deliver information through recorded messages or if they would prefer to speak with a live person. Except for males in Kandahar who were evenly split on the options, most respondents stated a preference for speaking with a live person rather than hearing a recorded message ( Table 7).

**Table 7.** Preferred way to receive health information through a mobile phone-based system, by sex and province, IDP youth Afghanistan 2017.

Media	Kandahar		Nangarhar		Takhar	
	Men (n=75) %	Women (n=74) %	Men (n=75) %	Women (n=76) %	Men (n=75) %	Women (n=75) %
Hear a recorded message	52.0	12.2	16.0	1.3	12.0	17.3
Speak with a live person	48.0	82.4	80.0	90.8	85.3	73.3
Don’t know	0.0	5.4	4.0	7.9	2.7	9.3

## Discussion

Results from this study add to limited information on the potential use of mobile phone technology to disseminate health-related information in fragile settings like Afghanistan. To our knowledge, this study is among the first to explore this topic among IDP youth.

Providing accurate, concise RMNCH information is important to the health of young men and women and a key step to improving uptake of RMNCH interventions. Such information has been traditionally delivered through health providers, and more recently, through lay persons trained as CHWs, consistent with reports from participants in this study. A key challenge with health care providers being the primary RMNCH information channel is limited access to such providers experienced in many low- and middle-income countries, including Afghanistan. Even when services are available, costs and perceived low quality can impede health service uptake ^12^. Women in Afghanistan have the added challenge of needing permission and money for payment from male relatives to obtain health services ^3^. These barriers can intensify for IDPs, particularly the recently-displaced, as they are unfamiliar with health facilities in their new location and some may fear discrimination based on IDP status ^23^.

Lay CHWs are an important health information source, particularly in more rural, remote settings, and findings from this study reflect the value participants place on CHWs as a RMNCH information channel. Notably, CHWs were named frequently in Kandahar, despite sampling most of the IDP youth from urban locations in that province, potentially reflecting individuals recently displaced from rural areas. However, persistent challenges such as competing demands and high turn-over among CHWs can limit their effectiveness to reach male and female youth in some settings ^24^. Moreover, for youth displaced to urban settings, CHWs are not a viable channel as the urban health system does not have a formal CHW cadre ^24^.

Mass media campaigns are another health information strategy with evidence for increasing health-related knowledge, attitudes, and to a lesser extent, health behaviors ^25– 27^. Regardless of effectiveness, use of media like print, radio and television requires reasonable access among the target audience. Our findings suggest that low literacy precludes using print media to disseminate health information, and RMNCH information delivered through radio and television would miss large numbers of youth in these IDP communities. Youth in Kandahar were more likely to report television use, likely reflecting the predominantly urban recruitment sites for this province and thus both access and acculturation to this channel among youth living in an urban location ^28^. Though we did not measure duration of displacement, anecdotal information indicated that some participants had been resettled for five years or more, potentially resulting in different norms around media use and health care access compare to those more recently displaced.

It is notable that some male youth reported need for RMNCH-related information, in some cases exceeding the proportion of female youth requesting the same information for certain topics. We posit this difference may reflect limited reliable information sources accessible to young men coupled with their awareness of becoming the primary health decision-maker upon marriage ^3^. Just under half of youth in this study were already married; thus, these topics are germane to this population. On-demand channels, as well as programming specifically targeting male IDP youth, are worthy of further investigation.

Although few participants spontaneously listed mobile phones as a preferred health information channel, most reported being likely to use a free mobile phone-based intervention. Given low literacy levels in Afghanistan, options for a mobile phone information system are limited to voice delivery, either through pre-recorded messages or speaking with a live person, with the latter strongly preferred by youth in this study ^20, 29^. With high reported mobile phone access among participants, such a system seems potentially feasible and likely accessible to more youth than other communication channels.

There is limited reported evidence on the use of messaging strategies for mobile phones other than SMS, and to date, there is no published evidence on the use of mobile phone-based health promotion interventions in Afghanistan ^14^. Authors of a 2017 systematic literature review on mHealth strategies in low- and middle-income countries found that most intervention strategies used SMS-based messaging, though a few incorporated social media apps, such as Facebook®, and one program in Papua New Guinea employed the use of voice messages and an interactive information hotline to promote youth-friendly sexual and reproductive health information for young people ages 15 to 24 ^14^. While studies and project reports have documented successful implementation of mobile health strategies, predominately SMS messaging, a 2019 review notes a number of important barriers, including telecommunications infrastructures, costs, literacy and language barriers, among others, that will require careful consideration, planning and assessment as future interventions are developed and evaluated ^30^. Designing and implementing a mobile phone-based RMNCH information program targeting youth must also consider potential challenges to reaching young people, particularly female youth. It will also need to be tailored to the information needs of subpopulations, which differ by geography, with substantial differences in education and literacy, in particular, among male IDP youth across provinces. Although mobile phone access among Afghan women is high and growing, social norms that require women to seek permission to use phones and privacy concerns when phones are shared within families are important considerations ^20^.

Geography, ethnic diversity, limited transportation infrastructure and insecurity make conducting population-based research in Afghanistan challenging. While this study provides useful information on health information sources and preferences of youth from three provinces, several limitations require interpreting findings with caution. The youth in these analyses self-identified as IDP during enrollment; however, we did not collect information on reasons for displacement and length of time in current residence. Thus, this sample of youth may be quite heterogenous by displacement characteristics. although efforts were made through use of a random-walk method to reduce sampling bias in this convenience sample, it remains a non-probability sample; ^31^ the extent to which findings reflect the broader population of IDP youth in these provinces is unknown. Information collected on exposure to media and health-related information was gathered through self-report and are subject to problems with recall.

Although findings from this study are exploratory, we believe that mobile phone-based programming, if appropriately marketed, presents a promising channel to reach IDP youth. Further research is needed to more thoroughly document feasibility, as well as to understand the most cost-effective mobile phone platforms to use. It will also be important to refine content for various RMNCH topics and channel selection to target specific segments of these sub-populations. We recommend that mobile phone-based RMNCH programming be developed and tested specifically for youth, with purposive inclusion of IDP populations in multiple settings to ensure cultural congruence and acceptability. The resulting interventions should be rigorously tested for cost-effectiveness to help guide national policy for youth programming amidst budgetary limitations and increasing insecurity.

## Data availability

### Underlying data

Harvard Dataverse. Databases for Formative Assessment of Knowledge, Attitudes, and Preferred Media for Reproductive Health Engagement among Selected Groups of Youth and Men in Afghanistan. https://doi.org/10.7910/DVN/QULMNN ^21^.

Underlying data are available in file “IDPYouthRMNCHMediaKAPSani_30APR19.tab”

### Extended data

Harvard Dataverse. Databases for Formative Assessment of Knowledge, Attitudes, and Preferred Media for Reproductive Health Engagement among Selected Groups of Youth and Men in Afghanistan. https://doi.org/10.7910/DVN/QULMNN ^21^.

The data collection instrument is attached to the final study report (file “AfghaM Male Youth Report_Final26MAR19.pdf”) as ANNEXE 2: IDP YOUTH MNCH COMMUNICATIONS SURVEY QUANTITATIVE COMPONENT.

Data are available under the terms of the Creative Commons Zero “No rights reserved” data waiver (CC0 1.0 Public domain dedication).
