# Supplementary material for: Getting health information to internally displaced youth in Afghanistan: can mobile phone technology bridge the gap?
Source: Gates Open Res. 2019 Oct 23;3:1483. Originally published 2019 Jun 4. [Version 2] doi: 10.12688/gatesopenres.13008.2 (PMC7459210; doi:10.12688/gatesopenres.13008.2)
Supplement: Peer review history

# Peer review: Reviewer response for version 2

**DOI:** 10.21956/gatesopenres.14222.r29387

**Published:** 2020-09-02

**Authors:** Tanvir Ahmed, Syed Jafar Raza Rizvi

**Affiliations:** 1 Statistical Analyst, Johns Hopkins Centre for Communication Program, Baltimore, MD, USA; 2 Department of Oncology and Metabolism, Faculty Of Medicine, Dentistry and Health, The University of Sheffield, Sheffield, UK

Introduction

 The introduction appeared to be lacking in building a coherent and logical argument. As the title reflects, the article is about understanding the scope of mobile phones in ensuring access to health information by the internally displaced youth in Afghanistan. The introduction should talk about two specific issues: firstly, about the access to health information by the internally displaced youth in Afghanistan. This should justify the breadth of the problem and the need for an intervention. And secondly, a snapshot of the evidence on the successful use of mobile phones to ensure access. This will justify the use of mobile phones for developing such intervention. Then the introduction should present the mobile phone landscape of Afghanistan and the internally displaced Afghani youth (at least the youth only) as the potential users. Once this is done, a final paragraph to knowledge gap (e.g. how much is known about the willingness and preference of the internally displaced Afghani youth regarding accessing health information through mobile phone) and then explain the aim/objective of the paper.

 Method

 I found the method section somewhat ambiguous. It appears that the study is a sub-group analysis of a bigger project called HEMAYAT. The main ambiguity is regarding sampling. It appears that the sampling technique was Systematic Random, yet the authors did not clarify that. Also, the reason(s) for the equal number of male and female in the sample was not clear. This is a very important dimension of health and technology research globally, gender divide. Therefore, how equal sampling represents the community needed to be clarified. The method section also needs to clarify the variables used in the analysis and the specific ethical approach (consent) in interviewing the minors.

 Result

 The main observation regarding the result section is:

- The reported frequencies (numbers and percentages) are simple frequencies. The authors could have easily performed nonparametric tests to measure the significance of the differences by gender or geography etc. Its is unclear why they didn’t do it. Also, results would have been more presentable if each table included three more columns (cumulative for all locations): all, male and female. And then went on to present the gender dichotomy by locations.
- In the first paragraph of the subhead ‘Health information and preferred information sources,’ I could not find the percentages reported in Table 5. Which table does this refer to? Also, there seems to be an effort to report ranking based on multiple responses (MR) variables. This needs to be corrected.
- The preference part (2 ^nd^ paragraph under the subhead ‘Health information and preferred information source’) was very interesting. It is not clear what measures were taken to triangulate or probe the respondents' preferences. Generally, without such an approach, the scope of any reported preference is very limited and sometimes unacceptable. The authors can look at ‘Contingent Valuation (CV)’ as one of such method for reference. This approach is based on game theory and is widely accepted for reporting preferences. Also, I could not find the 16.4% in Table 6 or anywhere.

Discussion

 I found the discussion is very unrelated to the reported result. It seems to be driven by literature review solely and assumptions. As a result, there is no central flow towards a central message. Also, the discussion section needs to refer back to the aim/objective of the paper to show the relevance of the findings or how much it has been achieved. In my opinion, the authors should rewrite the discussion as:

- Starting with a paragraph of the summary of the result and its relevance to the objective of the paper.
- Describe the context of young people and health through technology in Afghanistan in light of global evidence.
- Explain the limitation and what should be done to minimize.

As this flow was lacking, I did not understand how the authors were recommending developing the mobile phone-based intervention. In reality, this paper should have embarked on more rigour research on the demand, supply and technique of technology-based health information dissemination especially in geographically and resource-constrained contexts like Afghanistan.

Is the work clearly and accurately presented and does it cite the current literature?

Partly

If applicable, is the statistical analysis and its interpretation appropriate?

Partly

Are all the source data underlying the results available to ensure full reproducibility?

No

Is the study design appropriate and is the work technically sound?

Partly

Are the conclusions drawn adequately supported by the results?

No

Are sufficient details of methods and analysis provided to allow replication by others?

No

Reviewer Expertise:

Health System Research, Health and Technology, Maternal and Child Health, Healthcare Financing, Health Service Research, Complex Adaptive Systems, Mixed-Method Research etc.

We confirm that we have read this submission and believe that we have an appropriate level of expertise to confirm that it is of an acceptable scientific standard, however we have significant reservations, as outlined above.

# Peer review: Reviewer response for version 2

**DOI:** 10.21956/gatesopenres.14222.r29363

**Published:** 2020-08-27

**Authors:** Lianne Gonsalves

**Affiliations:** 1 Department of Sexual and Reproductive Health and Research including UNDP/UNFPA/UNICEF/WHO/World Bank Special Programme of Research, World Health Organization, Geneva, Switzerland

This is a very interesting read and covers phone access versus ownership among young people (an area where data is sorely needed). The study being conducted in Afghanistan makes it valuable as well, given (as the authors acknowledge) there is a lack of digital health-related literature from this country. 

 I applaud the authors for presenting results that aren't particularly promising with relation to digital access and appreciate that they do not attempt to overstate their findings (or push digital *too* hard). My comments are minor and mostly relate to how the Methods are presented.

**Introduction:** consider including a definition of IDP, for those not as familiar with it.

**Methods:**

[Overarching comment]:

 My understanding is that the data presented are youth-specific data collected as part of a larger cross-sectional formative survey of the general(?) population of youth and adult males (rather than just the IDP population). However, the Methods section, as currently presented, makes it appear as though there was different data collection methods for the general population, IDP-related population, youth-related parts of the IDP population.

 Example: the geographic areas that were selected, was this *only* for the IDP-related participants? Or was this part of the general sampling? Did random walk sampling only happen for IDP related participants or the broader sample?

 Following the first sentence of the Methods section, I would suggest reworking the rest of the section to begin with the relevant parts of the broad survey (e.g. when data was collected, how data was collected, the subject matter of the survey), including where it took place, and how many people were recruited. Subsequent paragraphs can narrow to the pieces specific to this sub-group analysis. At the very least, specify which pieces are specific to which populations, for example ‘In order to identify IDP participants specifically, in each province we conducted additional sampling using a random walk technique.’ (it’s currently difficult to tell whether the whole survey used random walk or just the IDP sub-sample).

[Additional specific comments]:

 Consider including how many people were participating in the entire survey.

**Results:**

 In general, please consider indicating for each section whether respondents were given options to choose from or asked to name things spontaneously – this is an important piece of context for interpreting the results (especially given that so few young people ‘spontaneously’ mentioned being interested in phones as sources of health information).

 Table 2: please clarify what is meant by ‘access’. Is this talking about whether the channel *is available* in the household, or whether the respondent perceived that they themselves could access it? My assumption is the latter, otherwise why would women respondents be in households (which many of which presumably also include men, given marriage numbers) that have significantly fewer phones than men respondents. (If this is actually the case, it’s worth commenting on).

**Discussion:**

 The authors acknowledge that cost is a concern for client-targeted digital interventions in general (and in relation to platform selection), but not specifically to the channels they propose either voice (which in many countries can be an expensive channel), or live hotline (which, apart from being an expensive channel also includes the human resource costs of having someone on the other end). It’s worth reflecting on this, possibly in the fourth paragraph from the end.

[This is my personal perspective, which the authors are not obliged to incorporate if they disagree with]:

 Finally, I disagree with the authors' final conclusions. My personal take is that the lack of phone access (and equity of access, for women in particular), and lack of spontaneous enthusiasm for a phone-based intervention (questions resulting in data presented in Figure 1 tend to be quite leading) means that digital *alone* may *not* be the most effective channel (in terms of cost, coverage, and/or uptake) to reach an already hard-to-reach population that does not have consistent access to any one channel. Having a campaign available through several channels (radio, CHW sensitization, in addition to digital, for example) and/or tied to existing health programming could help the authors avoid the pitfalls of stand-alone youth-targeted digital health interventions that others have fallen into.

Is the work clearly and accurately presented and does it cite the current literature?

Yes

If applicable, is the statistical analysis and its interpretation appropriate?

Yes

Are all the source data underlying the results available to ensure full reproducibility?

Yes

Is the study design appropriate and is the work technically sound?

Yes

Are the conclusions drawn adequately supported by the results?

Partly

Are sufficient details of methods and analysis provided to allow replication by others?

Partly

Reviewer Expertise:

Digital health, adolescent sexual and reproductive health

I confirm that I have read this submission and believe that I have an appropriate level of expertise to confirm that it is of an acceptable scientific standard, however I have significant reservations, as outlined above.

# Peer review: Reviewer response for version 2

**DOI:** 10.21956/gatesopenres.14222.r28121

**Published:** 2019-11-12

**Authors:** George C. Patton

**Affiliations:** 1 Department of Paediatrics, University of Melbourne, Melbourne, Victoria, Australia

The authors have responded to my comments with as much information as they are able to provide. I don't think they could do much more given the data they have available. 

 The paper makes a useful contribution to a topic about which we have known very little.

Is the work clearly and accurately presented and does it cite the current literature?

Partly

If applicable, is the statistical analysis and its interpretation appropriate?

Yes

Are all the source data underlying the results available to ensure full reproducibility?

Yes

Is the study design appropriate and is the work technically sound?

Partly

Are the conclusions drawn adequately supported by the results?

Partly

Are sufficient details of methods and analysis provided to allow replication by others?

No

Reviewer Expertise:

NA

I confirm that I have read this submission and believe that I have an appropriate level of expertise to confirm that it is of an acceptable scientific standard.

# Peer review: Reviewer response for version 1

**DOI:** 10.21956/gatesopenres.14116.r27473

**Published:** 2019-07-15

**Authors:** Nichole Stetten

**Affiliations:** 1 College of Public Health and Health Professions, University of Florida, Gainesville, FL, USA

This study highlights the need for reproductive, maternal, newborn and child health (RMNCH) interventions in Afghanistan. There is a need for RMNCH interventions to be targeted towards youth, aged 15 to 25 years old, which make up more than half of the Afghani population. Internally displaced persons (IDPs) within this population have even greater barriers to access maternal and child health care. Due to IDPs being a unique population and harder to reach, mobile technology could be used to bridge the access gap in health care. The study aimed to measure the current and preferred RMNCH information channels with an emphasis on exploring the feasibility of using mobile phone technology to reach the IDP youth population.

 The results of this study are important to the field especially as it covers a hard to reach population. Other strengths of the study are its large sample size over three provinces, with almost equal male and female participants within each province. Additionally, the methods show great cultural competency, which is crucial when conducting a research study within a community.  

 Current recommendations to the authors are listed below:

- Brief mention of where the survey data comes from is needed and the purpose of the overall project should be added. Looking at the references within this section, the data seems to come from the United States Agency for International Development (USAID)-funded Helping Mothers and Children Thrive in Afghanistan (HEMAYAT) project. Further explanation of this project would strengthen the paper and deepen understanding. 
- Formative research is primarily used for developing intervention strategies, materials and instruments. Commonly formative research approaches are developed using a theoretical framework. Methods should be expanded to include how this formative approach was developed. 
- The random walk technique used for sampling should be explained in further detail, including why this method was chosen over other methods. How does this method reduce bias? 
- In the methods the authors state that they were specifically targeting youth and adult men, but when looking in the recruitment section it appears, they are targeting men and women equally.
- Looking at the demographics of the study it looks as if there are significant differences in men in the Takhar province than the other two. While women were similar in demographics across the three provinces, men in the Takhar province had lower levels of educational attainment and low levels of literacy. This difference should be noted within the paper. Noting these differences not only with demographics but in the other sections of the analysis is important as certain provinces may have different wants/needs. If these populations are significantly different from each other then they may also have different needs within an intervention.
- The authors conclude that an RMNCH intervention is “potentially feasible” and “likely accessible to more IDP youth” than other forms of communication. The results appear to show low feasibility for this type of intervention, due to low literacy rates, smart phones being owned and controlled predominantly by males, and overall preferred sources of RMNCH coming directly from health care providers. Although many IDP youth own mobile phones there would be many barriers to overcome to implement a successful mobile intervention. A majority of participants reported being “likely to use a free mobile phone-based intervention,” this high rate of “intention to use” does not always translate into a behavior change. Actual feasibility conclusions should be drawn from a pilot study using an RMNCH intervention.

Is the work clearly and accurately presented and does it cite the current literature?

Yes

If applicable, is the statistical analysis and its interpretation appropriate?

Not applicable

Are all the source data underlying the results available to ensure full reproducibility?

Yes

Is the study design appropriate and is the work technically sound?

Yes

Are the conclusions drawn adequately supported by the results?

Partly

Are sufficient details of methods and analysis provided to allow replication by others?

Partly

Reviewer Expertise:

PhD in Public Health - Behavioral Science and Community Health

I confirm that I have read this submission and believe that I have an appropriate level of expertise to confirm that it is of an acceptable scientific standard, however I have significant reservations, as outlined above.

# Response

**Published:** 2019-10-21

**Authors:** Lisa Dulli

**Affiliations:** FHI 360, USA

Thank you for your thoughtful and detailed review. We've tried to address each concern separately below (reviewer comments in italics, responses in normal text):

*This study highlights the need for reproductive, maternal, newborn and child health (RMNCH) interventions in Afghanistan. There is a need for RMNCH interventions to be targeted towards youth, aged 15 to 25 years old, which make up more than half of the Afghani population. Internally displaced persons (IDPs) within this population have even greater barriers to access maternal and child health care. Due to IDPs being a unique population and harder to reach, mobile technology could be used to bridge the access gap in health care. The study aimed to measure the current and preferred RMNCH information channels with an emphasis on exploring the feasibility of using mobile phone technology to reach the IDP youth population.*

*The results of this study are important to the field especially as it covers a hard to reach population. Other strengths of the study are its large sample size over three provinces, with almost equal male and female participants within each province. Additionally, the methods show great cultural competency, which is crucial when conducting a research study within a community.*

*Current recommendations to the authors are listed below:*

- *Brief mention of where the survey data comes from is needed and the purpose of the overall project should be added. Looking at the references within this section, the data seems to come from the United States Agency for International Development (USAID)-funded Helping Mothers and Children Thrive in Afghanistan (HEMAYAT) project. Further explanation of this project would strengthen the paper and deepen understanding. *

Information on the project is added to the first sentence of the methods section with new content in italicized font on pp. 2-3:

“We conducted a sub-group analysis of survey data collected in a mixed-methods, cross-sectional, formative assessment *to inform programming for the United States Agency for International Development-funded Helping Mothers and Children Thrive in Afghanistan project*. ^21^ The assessment was designed to gather information from youth *15-25 years* and adult men *> 25 years of age* *to identify health information and service gaps related to RMNCH outcomes* *and inform content and channel selection to segments of these populations, including IDPs, for targeted programming*. For these analyses, we include data *from a convenience sample* of female and male IDP youth, ages 15 to 25 years. Youth *who self-identified as IDP* were recruited from households located in areas with large IDP populations in Kandahar, Nangarhar, and Takhar Provinces, selected based on their geographic and ethnic diversity. Survey data were collected March-July 2017 using a structured questionnaire administered through face-to-face interviews conducted by trained research assistants. Data used for these analyses include participant background information, household possession of media devices (i.e. television, radio and mobile phones), exposure to health information from various channels, desires for various types of health information and *preferred* channels of communication, as well as likelihood to use a free mobile phone-based system to receive health information.” 

 

- *Formative research is primarily used for developing intervention strategies, materials and instruments. Commonly formative research approaches are developed using a theoretical framework. Methods should be expanded to include how this formative approach was developed. *

While we agree that formative research can be driven by theory, it can also be driven by empirical evidence (or both) or practical need in the absence of empirical evidence. In this case, the project had a number of predetermined objectives (based on project design) and this assessment was used to identify gaps that needed to be addressed for those objectives in order to target interventions to improve health outcomes. We have added this content on pp. 2-3:

“We conducted a sub-group analysis of survey data collected in a mixed-methods, cross-sectional, formative assessment to inform programming for the United States Agency for International Development-funded Helping Mothers and Children Thrive in Afghanistan project. The assessment was designed to gather information from youth 15-25 years and adult men > 25 years of age to identify health information and service gaps related to RMNCH outcomes and inform content and channel selection to segments of these populations, including IDPs, for targeted programming.”

 

- *The random walk technique used for sampling should be explained in further detail, including why this method was chosen over other methods. How does this method reduce bias? *

The method was selected as an attempt to reduce sampling bias as compared to simple convenience sampling, although we acknowledge that it does not necessarily do so to a measurable extent; thus, we describe and treat these data as a convenience sample. Community mapping to generate a true probability sample is a time-intensive and costly endeavor, one that the project was unable to afford. Therefore, the option to enroll a convenience sample was chosen. However, random-walk sampling can help to reduce (though we do not know to what extent) sampling bias introduced by a non-random sampling method. Reference is given to the primary source that describes the method and we have provided further details on p. 3:

“Households in these communities were sampled using a random walk technique. ^22^ The approach used for this random-walk sample involved selecting a central community landmark, such as a mosque, then selecting one house at random to be the starting point. From there households were approached at regular intervals (e.g. every 3rd) Household selection continued in each area until reaching the requisite sample size.”

< > *In the methods the authors state that they were specifically targeting youth and adult men, but when looking in the recruitment section it appears, they are targeting men and women equally.*Looking at the demographics of the study it looks as if there are significant differences in men in the Takhar province than the other two. While women were similar in demographics across the three provinces, men in the Takhar province had lower levels of educational attainment and low levels of literacy. This difference should be noted within the paper. Noting these differences not only with demographics but in the other sections of the analysis is important as certain provinces may have different wants/needs. If these populations are significantly different from each other then they may also have different needs within an intervention. *The authors conclude that an RMNCH intervention is “potentially feasible” and “likely accessible to more IDP youth” than other forms of communication. The results appear to show low feasibility for this type of intervention, due to low literacy rates, smart phones being owned and controlled predominantly by males, and overall preferred sources of RMNCH coming directly from health care providers. Although many IDP youth own mobile phones there would be many barriers to overcome to implement a successful mobile intervention. A majority of participants reported being “likely to use a free mobile phone-based intervention,” this high rate of “intention to use” does not always translate into a behavior change. Actual feasibility conclusions should be drawn from a pilot study using an RMNCH intervention.*

We appreciate the reviewer’s comments; however, as noted in the discussion, if the intervention used voice messaging (either through live persons or voice recordings) then the literacy challenge would be addressed. Smartphones would not be needed for either a voice- or a text-based intervention, and we hope we have not given the impression that they would be required.

We agree that face-to-face client-provider interactions are ideal based on the preferred source of health information for most participants; however, access to such interactions is limited, for multiple reasons, which is why alternative, complementary strategies are required.

In terms of self-reported likelihood to use a hypothetical intervention, we absolutely agree. We’ve revised the text in the last paragraph to more explicitly support the need for documenting the actual feasibility of the approach on p. 9:

“Although findings from this study *are *exploratory, we believe that mobile phone-based programming, if appropriately marketed, presents a promising channel to reach IDP youth. Further research is needed to *more thoroughly document feasibility, as well as to understand the most cost-effective mobile phone platforms to use.* It will also be important to refine content for various RMNCH topics and channel selection to target specific segments of these sub-populations. We recommend that mobile phone-based RMNCH programming be developed and tested specifically for youth, with purposive inclusion of IDP populations in multiple settings to ensure cultural congruence and acceptability. The resulting interventions should be rigorously tested for cost-effectiveness to help guide national policy for youth programming amidst budgetary limitations and increasing insecurity.”

# Peer review: Reviewer response for version 1

**DOI:** 10.21956/gatesopenres.14116.r27302

**Published:** 2019-07-02

**Authors:** George C. Patton

**Affiliations:** 1 Department of Paediatrics, University of Melbourne, Melbourne, Victoria, Australia

This cross-sectional survey reports on a topic and youth group that has had very little attention in the literature. Certainly I’ve seen no similar report and as the authors rightly conclude, displaced youth in places like Afghanistan have been little studied. This population also poses quite a few challenges in surveys of this kind, as the authors again point out.

 The paper is generally well-written and the tables/figure are straightforward and clear. Inevitably there are methodological limitations, many of which the authors to flag. Some remained significant.

1. The sampling methods are not clear enough. Given the relatively small numbers, I am assuming this is not aspiring to be a representative sample of the target population. However, it isn’t clear how these participants were selected. No details are given of response rates, including characteristics of non-responders. These details are important.
2. One problem in interpreting the findings is the definition of internally displaced. The authors do mention this in the discussion as a limitation but it seems that these groups are actually young people living in areas where there are high levels of internal displacement? I was clear if they were displaced themselves – if not perhaps the title of the paper should be changed?
3. It would be very good if the authors could elaborate on reasons for internal displacement of youth in Afghanistan. I’m assuming that conflict and unrest is one factor but there are a range of others that are very significant: marriage in girls; employment in boys; and there may be a range of other reasons as well e.g. access to education. This is important for the reader to understand.
4. The conclusion that the authors draw is that there would be scope for communicating with males and to a lesser extent females through mobile phones to provide health information. Given youth preferences, this would ideally be a system with a real person at the other end of the phone line. Could the authors provide some examples of where this has been rolled out, ideally with information on coverage and costings, with some discussion as to whether this might be affordable and scalable in the context of Afghanistan?
5. The researchers seemed quite dismissive of mass media strategies for providing information to youth. Yet there are some very good examples of using mass media and low and middle income countries to provide information on sexual and reproductive health. It would be good if the authors could go back and re-examine this literature as mass media do provide an alternative platform for delivery of information at scale – the costs of doing so are likely to be quite a lot lower than individualised manning of telephone lines.

Is the work clearly and accurately presented and does it cite the current literature?

Partly

If applicable, is the statistical analysis and its interpretation appropriate?

Yes

Are all the source data underlying the results available to ensure full reproducibility?

Yes

Is the study design appropriate and is the work technically sound?

Partly

Are the conclusions drawn adequately supported by the results?

Partly

Are sufficient details of methods and analysis provided to allow replication by others?

No

Reviewer Expertise:

Developmental Epidemiology

I confirm that I have read this submission and believe that I have an appropriate level of expertise to confirm that it is of an acceptable scientific standard, however I have significant reservations, as outlined above.

# Response

**Published:** 2019-10-21

**Authors:** Lisa Dulli

**Affiliations:** FHI 360, USA

We thank the reviewer for his constructive feedback on this manuscript and submit the revised version along with responses to critiques.

Reviewer input is in italicized font and we provide our response and the relevant changes after each critique.

*This cross-sectional survey reports on a topic and youth group that has had very little attention in the literature. Certainly, I’ve seen no similar report and as the authors rightly conclude, displaced youth in places like Afghanistan have been little studied. This population also poses quite a few challenges in surveys of this kind, as the authors again point out. The paper is generally well-written and the tables/figure are straightforward and clear. Inevitably there are methodological limitations, many of which the authors to flag. Some remained significant.*

*1) The sampling methods are not clear enough. Given the relatively small numbers, I am assuming this is not aspiring to be a representative sample of the target population. However, it isn’t clear how these participants were selected. No details are given of response rates, including characteristics of non-responders. These details are important.*

This is not a representative sample, as noted in the limitations of the original manuscript. We have provided additional details on sampling strategy on pp. 2-3 (italicized text below):

“We conducted a sub-group analysis of survey data collected in a mixed-methods, cross-sectional, formative assessment *to inform programming for the United States Agency for International Development-funded Helping Mothers and Children Thrive in Afghanistan project*. ^21^ The assessment was designed to gather information from youth *15-25 years* and adult men *> 25 years of age* to *identify health information and service gaps related to* RMNCH outcomes *and inform content and channel selection to segments of these populations, including IDPs, for targeted programming*. For these analyses, we include data *from a convenience sample* of female and male IDP youth, ages 15 to 25 years. Youth *who self-identified as IDP* were recruited from households located in areas with large IDP populations in Kandahar, Nangarhar, and Takhar Provinces, selected based on their geographic and ethnic diversity. Survey data were collected March-July 2017 using a structured questionnaire administered through face-to-face interviews conducted by trained research assistants. Data used for these analyses include participant background information, household possession of media devices (i.e. television, radio and mobile phones), exposure to health information from various channels, desires for various types of health information and preferred channels of communication, as well as likelihood to use a free mobile phone-based system to receive health information.

Geographic areas were selected based on guidance from UNHCR, the Ministry of Refugees and Repatriation, and the Danish and the Norwegian Refugee Committees, who have pre-existing relationships with IDP communities and their leaders. Households in these communities were sampled using a random walk technique. ^22^ Household selection continued in each area until reaching the requisite sample size. Verbal informed consent was obtained from eligible youth interested in participating then a structured questionnaire was administered by sex-matched data collectors in a private setting within the house.”

and response rates on p. 4:

“A total of 462 IDP youth were recruited to participate in the survey. *Ten of the 462 declined participation (2.2%); one person consented, but refused to answer the remaining survey questions and data from one further participant was dropped because of incomplete interview, leaving* 450 total study participants in Kandahar, Nangarhar, and Takhar Provinces including 225 male and 225 female youth. *Characteristics of non-responders are not available as once a person declined to participate, no further information was recorded*.”

*2) One problem in interpreting the findings is the definition of internally displaced. The authors do mention this in the discussion as a limitation, but it seems that these groups are actually young people living in areas where there are high levels of internal displacement? I was clear if they were displaced themselves – if not perhaps the title of the paper should be changed?*

Although we did not collect additional information on displacement characteristics, eligibility was constrained to youth who self-identified as IDPs. Data collectors asked the youth if they were internally displaced before proceeding to the informed consent process. This information has been added to the manuscript on p. 3:

“For these analyses, we include data from a convenience sample of female and male IDP youth, ages 15 to 25 years. Youth who self-identified as IDP were recruited from households located in areas with large IDP populations in Kandahar, Nangarhar, and Takhar Provinces, selected based on their geographic and ethnic diversity.”

*3) It would be very good if the authors could elaborate on reasons for internal displacement of youth in Afghanistan. I’m assuming that conflict and unrest is one factor but there are a range of others that are very significant: marriage in girls; employment in boys; and there may be a range of other reasons as well e.g. access to education. This is important for the reader to understand.*

As noted by UNESCO, “Guiding Principles on Internal Displacement set by of Office for the United Nations High Commissioner for Refugees (UNHCR) holds internally displaced persons to be "persons or groups of persons who have been forced to flee, or leave, their homes or places of habitual residence as a result of armed conflict, internal strife, and habitual violations of human rights, as well as natural or man-made disasters involving one or more of these elements, and who have not crossed an internationally recognised state border." ( http://www.unesco.org/new/en/social-and-human-sciences/themes/international-migration/glossary/ displaced-person-displacement/). However, it could certainly be argued that the line between forced and unforced migration can be blurry. 

We noted in the discussion section, a limitation of the study is that we did not collect information from participants on reasons for displacement. We do, however, report in the second paragraph of the background section, main drivers for internal displacement (forced migration) in Afghanistan, which include conflict, natural disasters and poverty, as documented through other research; youth move with their families as the family unit tends to stay intact in situations resulting in displacement. We did not explore reasons for voluntary migration in detail.  

Please see the text on p. 1:

“Years of armed conflict, natural disasters like drought and flooding, and widespread poverty have led to large-scale migration of intact family units both within and from Afghanistan. ^9-11^”

*4) The conclusion that the authors draw is that there would be scope for communicating with males, and to a lesser extent, females through mobile phones to provide health information. Given youth preferences, this would ideally be a system with a real person at the other end of the phone line. Could the authors provide some examples of where this has been rolled out, ideally with information on coverage and costings, with some discussion as to whether this might be affordable and scalable in the context of Afghanistan?*

To date, there is no published evidence on the use of mobile phones for health promotion interventions in Afghanistan. Based on a search of the current literature, only one study has been published on the topic from Afghanistan - a descriptive study exploring women’s perceptions around using mobile phones to promote maternal and child health. There is very little in the published literature on the effectiveness of health promotion programs that employ voice messaging or live interactive phone calls in LMIC, and even less on costs. Many programs are currently being implemented, but the evidence lags behind. A number of protocols examining similar interventions have been published, but studies have not yet been completed. Of note, the HEMAYAT project, which led this formative research, is currently piloting a voice messaging intervention to promote RMNCH among pregnant and postpartum women. However, as with many of these interventions, the work is still underway. These limitations in the current literature are noted on p. 8:

“There is limited reported evidence on the use of messaging strategies for mobile phones other than SMS, and to date, there is no published evidence on the use of mobile phones in health promotion interventions in Afghanistan. ^14^ Authors of a 2017 systematic literature review on mHealth strategies in low- and middle-income countries found that most intervention strategies used SMS-based messaging, though a few incorporated social media apps, such as Facebook, and one program in Papua New Guinea employed the use of voice messages and an interactive information hotline to promote youth-friendly sexual and reproductive health information for young people ages 15 to 24. ^14^ Although studies and project reports have documented successful implementation of mobile health strategies, SMS messaging predominately, a 2019 review notes a number of important barriers, including telecommunications infrastructures, costs, literacy and language barriers, among others, that will require careful consideration, planning and assessment as future interventions are developed and evaluated. ^30^”

*5) The researchers seemed quite dismissive of mass media strategies for providing information to youth. Yet there are some very good examples of using mass media and low- and middle-income countries to provide information on sexual and reproductive health. It would be good if the authors could go back and re-examine this literature as mass media do provide an alternative platform for delivery of information at scale – the costs of doing so are likely to be quite a lot lower than individualised manning of telephone lines.*

Our aim was not to be dismissive of mass media, but rather it was to place our findings in the context of what is known about reaching youth with health information in this particular setting. There are indeed examples of mass media to influence health-related knowledge and attitudes, and to a lesser extent, health-related behaviors in other countries. We have revised our summary on p. 8 to read

*“Mass media campaigns are another health information strategy with some evidence for increasing health-related knowledge and attitudes, and to a lesser extent, health-related behaviors. service use, though the evidence is weak, and effectiveness for changing health behaviors is not well documented.”*

However, our findings indicate that access to traditional mass media channels (e.g. radio and television) is limited among our study participants and varies considerably across provinces. In Takhar, for example, fewer than one in four of men and women had access to either radio or television, thus limiting their potential effectiveness, hence the need to identify other communication channels.
